# Companion animal and equine clinical research: a Nordic perspective

**DOI:** 10.1186/s13028-024-00787-1

**Published:** 2025-01-06

**Authors:** Bodil Ström Holst, Alejandro Engelmann, Gittan Gröndahl, Lotta Gunnarsson, Anita Haug Haaland, Anna Hielm-Björkman, Lars Moe, Marie Rhodin, Henrik Rönnberg, Marie Stråhle, Ylva Toljander, Annemarie Thuri Kristensen, Malin Hagberg Gustavsson

**Affiliations:** 1 Department of Clinical Sciences, Faculty of Veterinary Medicine and Animal Science, Swedish University of Agricultural Sciences, PO Box 7054, 750 07 Uppsala, Sweden; 2 SLU University Library, Swedish University of Agricultural Sciences, PO Box 7071, 750 07 Uppsala, Sweden; 3 Swedish Veterinary Agency (SVA), 751 89 Uppsala, Sweden; 4 Department of Companion Animal Clinical Sciences, Faculty of Veterinary Medicine, Norwegian University of Life Sciences, 1433 Ås, Norway; 5 Department of Equine and Small Animal Medicine, Faculty of Veterinary Medicine, University of Helsinki, PO Box 57, 00014 Helsinki, Finland; 6 Department of Animal Biosciences, Faculty of Veterinary Medicine and Animal Science, Swedish University of Agricultural Sciences, PO Box 7036, 750 07 Uppsala, Sweden; 7 Department of Veterinary Clinical Sciences, University of Copenhagen, Copenhagen, Denmark; 8 Present Address: Department of Animal Biosciences, Faculty of Veterinary Medicine and Animal Science, Swedish University of Agricultural Sciences, PO Box 7036, 750 07 Uppsala, Sweden

**Keywords:** Bibliometry, Cat, Clinical, Dog, Equine, Horse, Research, Veterinary

## Abstract

**Background:**

The societal value of cats, dogs and horses is high, and the companion and sport animal health care sector is growing. Clinical research concerning cats, dogs and horses is crucial for the development of evidence-based medical care that benefits animals and their owners, and has implications for human and environmental health from a One Health perspective. Basic information on companion animal and equine research enables more directed measures to improve conditions for research within the area. The aim of the present study was to describe Nordic companion animal and equine clinical research from 2010 to 2019, including bibliometrics, human resources and funding.

**Results:**

There were 2 042 published research publications originating from Nordic countries on cats (n = 282), dogs (n = 1 086), and horses (n = 781) from 2010 to 2019. The majority (83%) of the publications came from the four Nordic universities with veterinary programs. Seven percent of the publications were collaborations between two or more Nordic universities. Approximately 18% of the PhD theses (178 out of 970) from veterinary faculties or corresponding units concerned these species, most of them dogs (n = 86), followed by horses (n = 64), cats (n = 15) or a combination of these species (n = 13). The scientific areas cardiology, infectious diseases, reproduction, and surgery were prominent for all three species. A large proportion of grants were received from small- to medium-sized funding bodies, mainly funding running costs and only to a limited degree salaries. During 2010–2019, costs for veterinary and other services for cats and dogs steadily increased. The growth of the veterinary healthcare sector was not reflected in an increasing number of clinical research publications, for which no increase was seen after 2014.

**Conclusions:**

Despite a high societal value of the species, veterinary clinical research on sports and companion animals has not increased, in contrast to the veterinary healthcare sector. Activities stimulating the research area, e.g. funding bodies enabling coverage of salaries, are needed. The development of Nordic veterinary clinical care may benefit from strengthened research cooperation between countries.

## Background

Companion animals and horses play important roles in society, for instance, by providing companionship, promoting health and well-being, contributing to the economy, preserving cultural heritage, and fostering social connections (e.g. [1–14]). Their presence enriches the lives of individuals and communities, making them valuable members of our society.

Although not all clinical research is of direct relevance to clinical practice, clinical research benefits the well-being and welfare of these animals and is key to support evidence-based development of their diagnostics, treatments and preventive care, and is therefore important to the general society with broader implications for human health. Unfortunately, research funding sources and levels appear limited and the increasing societal and economic importance call for securing research funding for these important areas of veterinary medicine to benefit animal and human health. To support this the following work documents research activities and funding sources for the Nordic countries 2010–2019.

The societal value of cats and dogs is receiving increasing attention [15]. In the Nordic countries, 25–40% of households include companion animals, often regarded as family members [3, 16–18]. Attributes such as companionship and love are related to cats and dogs [2] reflecting strong relationships contributing to human well-being. Approximately 80% of dog owners describe the main function of their dogs as companion animals [19]. During the COVID-19 pandemic, pet owners were reported to have improved mental wellbeing and reduced anxiety due to support from their cats and dogs [6, 11], although contrasting results were also reported [20]. Cats, dogs and horses are beneficial from a One Health perspective and as contributors to public services. They are models for diseases they have in common with people, [9, 12, 13, 21–25], may reduce the risk of childhood asthma and atopy [24, 26, 27], and in older adults, dogs have a positive effect on physical activity and sleep [12]. Companion animals are part of our work force, and in addition to police, military and guide dogs may benefit the rehabilitation of children [8], be reading dogs [5], and scent-detecting dogs [28].

Dogs and cats are increasing in number. In Norway, there has been an estimated 25% increase in the number of dogs from 2001 to 2012, and the number of pet cats increased 50%, from 535 000 to 800 000, between 2001 and 2016 [29]. Many cats and dogs reach an old age, and with increasing age require more veterinary care [19, 30, 31]. Many pet owners expect advanced care for their cats and dogs [32], and veterinary care in the Nordic countries is professionalised, with satisfied clients [33]. A large proportion of cats and dogs in the Nordic countries are covered by animal insurance. Sweden is the European country with the highest proportion of insured pets [34]. In Sweden, the proportion of insured dogs is estimated to be 60–80%, and the proportion of insured horses only slightly lower [34–36], whereas the proportion of insured cats is approximately 25% [34].

This increasing population of cats and dogs, together with owner expectations and the development of veterinary medicine [32], has stimulated the growth of the companion animal healthcare sector. In Norway, the number and size of clinics and veterinary health services provided have increased [37] from 30 companion animal clinics in 1987 to 520 in 2022 [38]. In Sweden, household spending on pets has surged, rising over 200% from 1993 to 2014, a much greater increase than the 29% rise in consumer price index during the same period [3]. In 2017, the revenue of the cat and dog sector in Sweden was approximately 16 billion SEK, with veterinary care accounting for almost 25% [3]. The net revenue for veterinary limited companies in Sweden increased by 38% between 2013 and 2016, to approximately 4.4 billion SEK [34]. The 155 veterinary clinics that were members of the Swedish Animal Healthcare sector of the Swedish Federation of Green Employers had 1.2 million client visits in 2019 [33].

Horses are both sports animals and beloved companions, representing a several-fold higher economic investment and risk compared to dogs. In Sweden, the number of horses has evolved from approximately 90 000 in 1979 to 355 000 in 2016 [39]. The price of an ordinary pleasure horse in Sweden in 2022 was 30 000–70 000 SEK, and an estimated cost for maintaining a horse was 7 000 SEK/month. In 2019, Swedish equine industry revenue was approximately 30 billion SEK per year, including the revenue of betting [40]. Approximately 80% of Swedish horse owners use their horses for leisure, and one-third of these participate in horse shows [41]. The largest horse sports in Sweden in 2022 were show jumping, harness racing, dressage, and eventing [41, 42].

Equestrian sport is the second largest sport in Sweden, with 0.5 million riders, and one of the largest sports for people with disabilities [43, 44]. Horse riding has a positive effect on e.g. children with autism [7], stroke rehabilitation in adults [4], identity construction in disabled people [10] and quality of life in people with neurological disorders [14].

The Nordic countries, except Iceland, each have had one single national university for research-based veterinary education and, at some universities, of veterinary nurses and technicians. Veterinary educations are offered at the University of Copenhagen (UCPH), Denmark; the University of Helsinki (UH), Finland; the Norwegian University of Life Sciences (NMBU) and the Swedish University of Agricultural Sciences (SLU).

During the past decade, capital fund-owned companion animal health care providers have changed the private practice landscape significantly. This has created new opportunities for clinical research on cats, dogs and horses, both as collaborations with academia and as company-driven research efforts. However, collated information on companion animal and equine research across Nordic countries is lacking.

The recent development of the companion animal sector is reflected in household costs for veterinary and other services and for purchase of the animals, their feed, equipment and accessories. Information on the development of such costs is available for cats and dogs in Sweden and allows comparisons with the development of clinical research to point to whether research activities are on par with the growing importance of the sector.

Basic information on companion animal and equine research enables more directed measures to improve conditions for research within the area. The aim of the present study was to describe Nordic companion animal and equine clinical research from 2010 to 2019, including bibliometrics, human resources and funding.

## Methods

### Bibliometrics

Bibliometric analyses were conducted by the SLU University Library. A search was performed in the Web of Science Core Collection, Indexes—Science Citation Index Expanded, Social Sciences Citation Index, Arts & Humanities Citation Index (Clarivate), and research articles (including review articles) published from 2010 to 2019 with at least one author with an address in a Nordic country were included. The search strategy used TS (topic) = [(cat or cats or feline or kitten or kittens) or (dog or dogs or canine or puppy or puppies or bitch or bitches) or (horse or horses or equine or mare or mares or stallion or stallions or foal or foals or pony or ponies or racehorse or racehorses)] in combination with CU (country/region) = (sweden or denmark or finland or norway or iceland).

All titles were scrutinized in two steps by two researchers (MHG and BSH), first for identification of the chosen animal species (removing articles such as “Algebraic new foundations”—mentioning MLCat in the abstract), and then to distinguish which of these publications should be included as “clinical research”. When needed, the articles’ abstracts and introductions were studied. Clinical research was defined as aiming at preventing, diagnosing, treating, or prognosticating diseases, as well as translating basic science research findings to clinical use. A wide spectrum of articles was included, e.g., physiology studies and some genetic studies. Articles in which animals were used as models for human disease with no intention of contributing to animal health were excluded (e.g. “Alveolar bone remodeling after tooth extraction in irradiated mandible: An experimental study with canine model”), as well as articles without clinical relevance, e.g., palaeontology studies (e.g. “Sexing Viking Age horses from burial and non-burial sites in Iceland using ancient DNA”). The final dataset was used for descriptive statistics and network analyses.

For an overview of the most prominent research topics in the publications, network visualizations of term co-occurrences were created using the software VOSviewer, version 1.6.16 [45]. Terms, i.e., noun phrases, were extracted from the titles and abstracts of the publications using natural language algorithms [46]. A VOSviewer thesaurus file was used for preprocessing of the extracted terms to merge synonyms (e.g., “anesthesia” and “anaesthesia”) and to remove frequent terms related to person, animal, time/place as well as general terms regarding scientific analysis and reasoning (e.g., “calculation”, “hypothesis”).

For each of the species cat, dog, and horse, network visualizations were created from terms occurring in a minimum of 4 publications (normalization method: association strength; attraction = 2, repulsion = 0). The 60% most relevant terms, based on their VOSviewer relevance score [46], were included in the term network (cat n = 112; dog n = 635; horse n = 520). The distance between two terms in the network reflects the relatedness of the terms. Node size represents term frequency (binary counting).

### Doctoral theses

The university libraries of NMBU, SLU, UCPH and UH were asked for lists of all doctoral theses concerning cats, dogs and horses 2010–2019 from the Department of Veterinary and Animal Sciences and Department of Veterinary Clinical Sciences at UCPH; the Faculty of Veterinary Medicine at UH and NMBU, and the Faculty of Veterinary Medicine and Animal Science at SLU.

### Human resources

The mean numbers of clinical professors and senior lecturers with research focused on cats, dogs and/or horses at the Department of Companion Animal Clinical Sciences (at NMBU), Department of Clinical Sciences (at SLU), Department of Veterinary Clinical Sciences (at UCPH), and the Department of Equine and Small Animal Medicine (at UH) in January 2021 were collected from representatives of the clinical departments at the respective universities.

The total number of European Veterinary Specialists^™^, awarded by EBVS^®^ (European Board of Veterinary Specialization) in Norway, Sweden, Denmark and Finland, registered as of January 2021 was collected from the EBVS^®^ website [47], as well as the number of European Veterinary Specialists^™^ in colleges that were most closely linked to clinical research in cats, dogs and horses. Defined as most closely linked to clinical research in cats, dogs and horses were the European Colleges of Animal Reproduction, Equine Internal Medicine, Veterinary Anaesthesia and Analgesia, Veterinary and Comparative Nutrition, Veterinary Clinical Pathology, Veterinary Dermatology, Veterinary Diagnostic Imaging, Veterinary Internal Medicine-Companion Animals (internal medicine, cardiology, oncology), Veterinary Neurology, Veterinary Ophthalmologists, Veterinary Pathologists, Veterinary Pharmacology and Toxicology, Veterinary Surgeons, Veterinary Sports Medicine and Rehabilitation, Veterinary Dental College, and the European Veterinary Parasitology College.

### Funding of veterinary clinical research

Representatives from the clinical departments at the respective universities contributed information on the most common funding bodies.

From one governmental funding body, the Swedish Research Council Formas, lists with all applications to the annual open call (including research projects, research projects for early-career researchers and mobility grants for early-career researchers) from 2010 to 2019 were obtained. All applications concerning dogs, cats and horses were compiled, and the proportions of granted and rejected applications were calculated.

Information was also directly obtained from the Agria and Swedish Kennel Club (SKK) Research fund, which is open to researchers from Denmark, Finland, Norway, Sweden and Germany.

### Household costs

To obtain a measure of the development of the sector, household costs were investigated, with Sweden as an example. Data on costs for “veterinary and other services for animals” and “companion animal feed and equipment” as well as information on the development of the consumer price index (CPI) were obtained from Statistics Sweden (www.scb.se). These statistics are based on data on registered persons that the Swedish Tax Agency supplies to Statistics Sweden. The CPI measures the average price trend for the entire private domestic consumption based on prices consumers pay and is the standard measure of compensation and inflation calculations in Sweden.

## Results

### Total number of peer reviewed publications

**Fig. 1:**
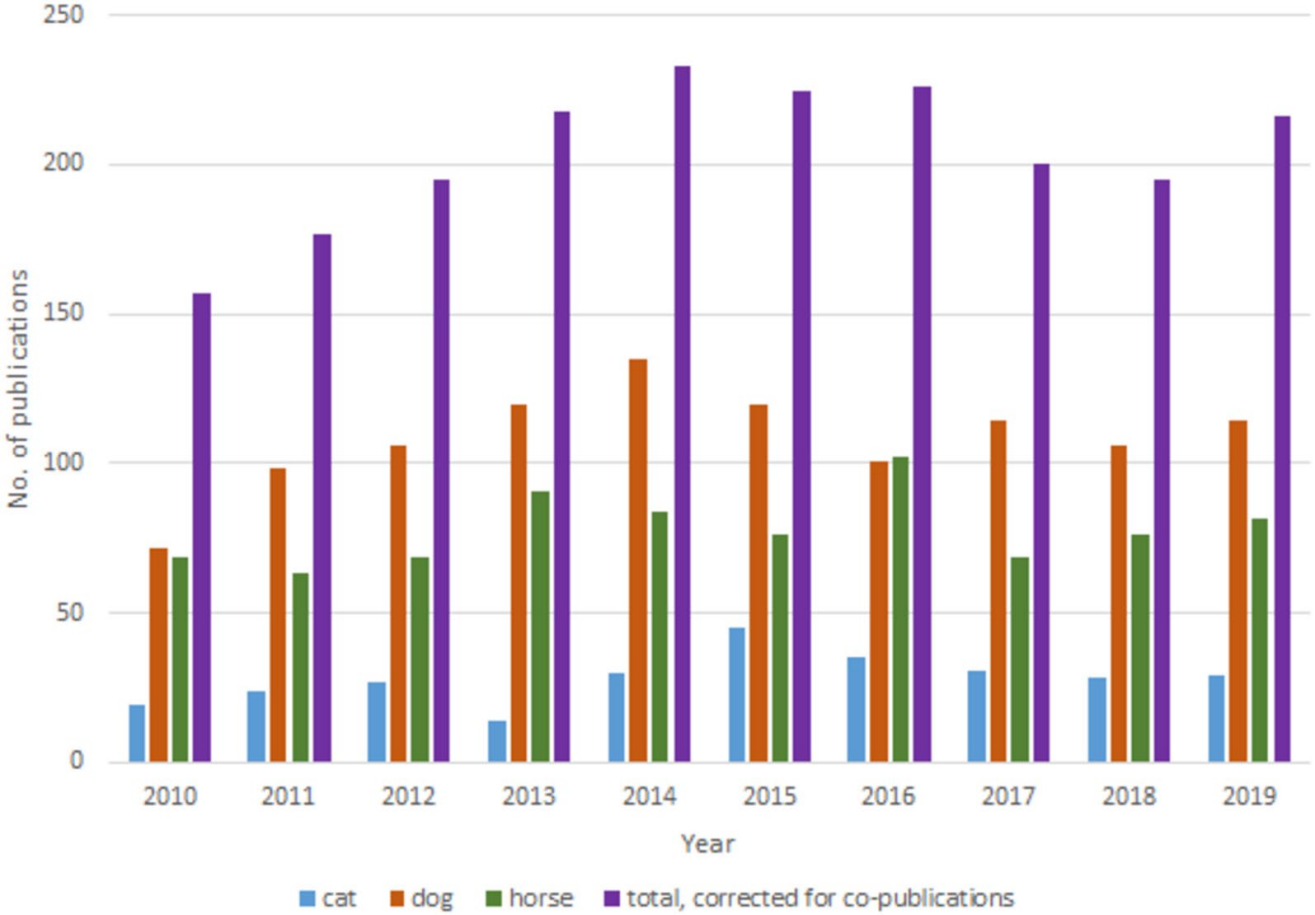
Clinical articles on cats, dogs and horses 2010–2019. Number of clinical articles on cats, dogs and horses, including total numbers corrected for co-publications, per year 2010–2019

The initial search resulted in 6 201 publications, out of which 2 972 concerned the relevant species. The total number of clinical canine, feline or equine publications in the Nordic countries from 2010 to 2019 was 2 042, 69% of the 2 972 publications that concerned these species. Of these, the majority, 53%, concerned dogs (n = 1086), followed by horses (38%, n = 781) and cats (14%, n = 282) (Some publications concerned more than one species, Table 1). For dogs, publications increased from 2010 and reached the highest number in 2014; for cats, 2015 was the year with the most publications, and in 2016, the number of publications for horses was the highest. An increase in the number of publications per year was noted from 2010 to 2014 and then reached a plateau (Fig. 1).

**Table 1:** Veterinary clinical research publications on cats, dogs and horses 2010–2019

University	Total (Total number of publications in country)	With authors from more than one of the universities %	Cat	Dog	Horse
NMBU (Norway)	221 (267)	62 (28)	26	124	75
SLU (Sweden)	664 (897)	105 (15)	89	326	268
UCPH (Denmark)	565 (647)	81 (14)	77	300	236
UH (Finland)	391 (458)	29 (7)	44	256	99
All universities corrected for coauthorship	1691 (Nordic countries total 2042)	127 (7)	282 (88 multiple species)	1086 (97 multiple species)	781 (20 multiple species)

Numbers of veterinary clinical research publications on cats, dogs and horses with authors affiliated with the Nordic universities during the time period 2010–2019. Because some publications concerned more than one species, the sum of publications on cats, dogs and horses does not agree with the total

*NMBU* Norwegian University of Life Sciences, *SLU* Swedish University of Agricultural Sciences, *UCPH* University of Copenhagen, and *UH* University of Helsinki

The four Nordic universities, in order, SLU (33%, n = 664), UCPH (28%, n = 565), UH (14%, n = 291) and NMBU (11%, n = 221), authored the majority of the publications. The species publication distribution of articles was similar for SLU and UCPH, with the majority concerning dogs (49% and 53%, respectively), almost as many concerning horses (40% and 42%, respectively), and fewest concerning cats (both 13%; Table 1). The dominance for articles concerning dogs was strongest (65%) for UH, with 25% horse articles and 11% cat articles. At NMBU, the corresponding figures were 56% for dogs, 34% horses and 12% cat articles (Table 1). Publications with authors affiliated with the two largest capital-fund owned companion animal health care providers, AniCura (from 2015) and Evidensia (from 2016), mainly concerned dogs (Table 2).

**Table 2:** Publications authored by AniCura (since 2015) and Evidensia (since 2016) to, and including, 2019

Company	Total publications	Cat	Dog	Horse	Copublications with Nordic universities
Evidensia	53	7	38	11	35
AniCura	23	6	17	0	17

### Collaborative peer-reviewed publications with Nordic or other international coauthorships

Out of the 127 Nordic copublications, 77 concerned dogs, 47 horses and 6 concerned cats. Of these, three articles included more than one species. The collaboration between UCPH and SLU resulted in most co-authored articles (n = 60), followed by NMBU-SLU (n = 44) and UCPH-NMBU (n = 24).

For dogs, the collaborative work resulting in the largest number of publications came from UCPH-SLU (35 articles), followed by NMBU-SLU (n = 27) and UH-SLU (n = 19). For horses, the collaborative work resulting in the largest number of publications came from UCPH-SLU (24 articles), followed by NMBU-SLU (n = 17) and UCPH-NMBU (n = 8). Only two publications concerned feline clinical research.

The major overall collaborative partners within canine, feline and equine clinical research were for NMBU: SLU (44 articles), UCPH (24) and University of Oslo (21); for SLU, they were Uppsala University (90 articles), UCPH (60) and NMBU (44); for UCPH, they were SLU (60 articles), University of Kentucky (33) and University of London (including Royal Veterinary College (30)); and for UH, the major collaborative partners were Folkhälsan Research Center (31 articles), Finnish Food Authority (24) and University of Turku (24).

### Prominent research areas

**Fig. 2:**
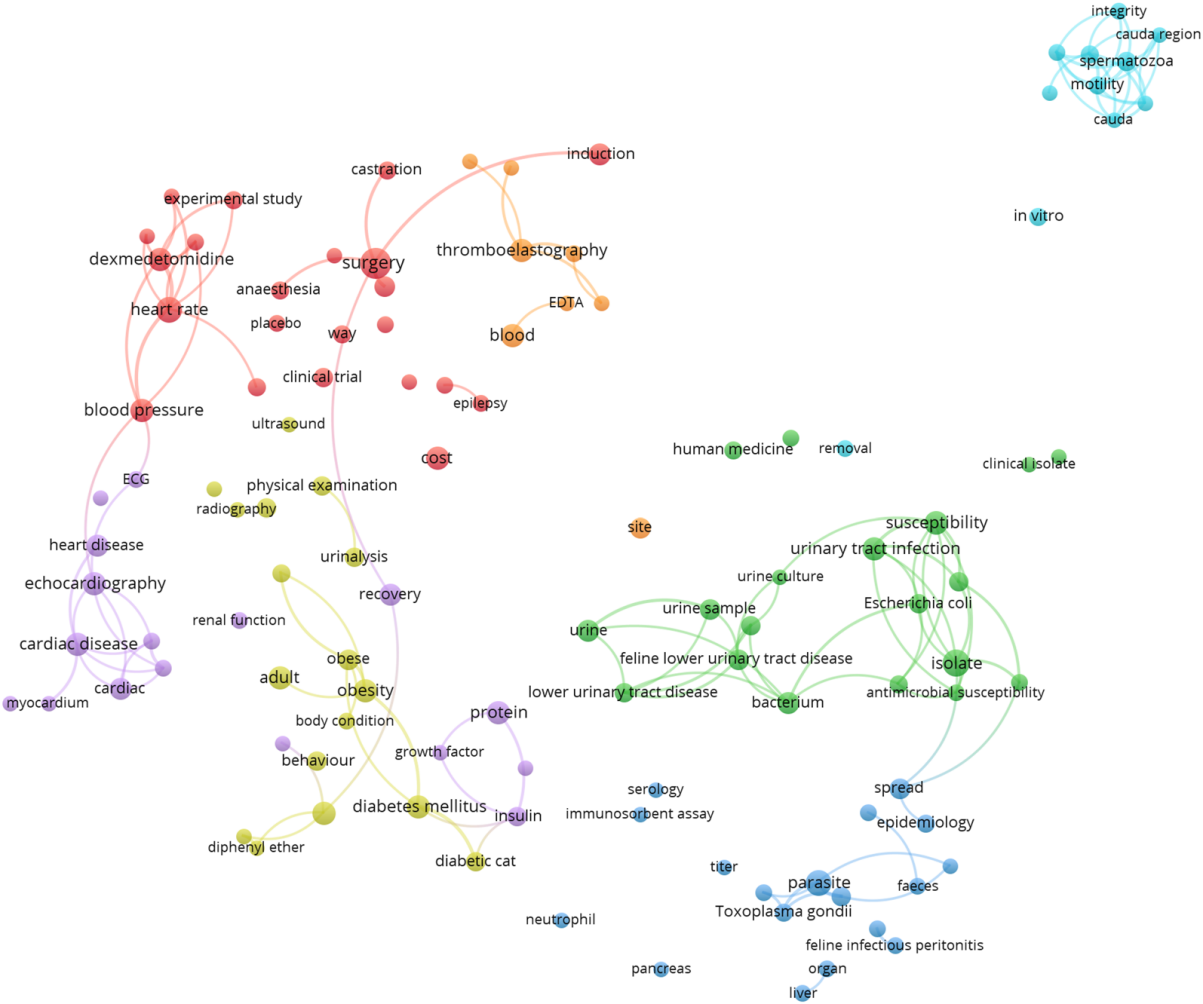
Term maps of the fields of clinical research on cats in the Nordic countries. The networks are based on term co-occurrences in the titles and abstracts of published clinical articles. The distance between two terms in a map reflects the relatedness of the terms, and colours represent clusters of related terms. The networks were created from terms occurring in a minimum of four articles. The 60% most relevant terms, based on their VOSviewer relevance score, were included in the maps (n = 112). Links are shown for pairs of terms which co-occur in at least three articles

**Fig. 3:**
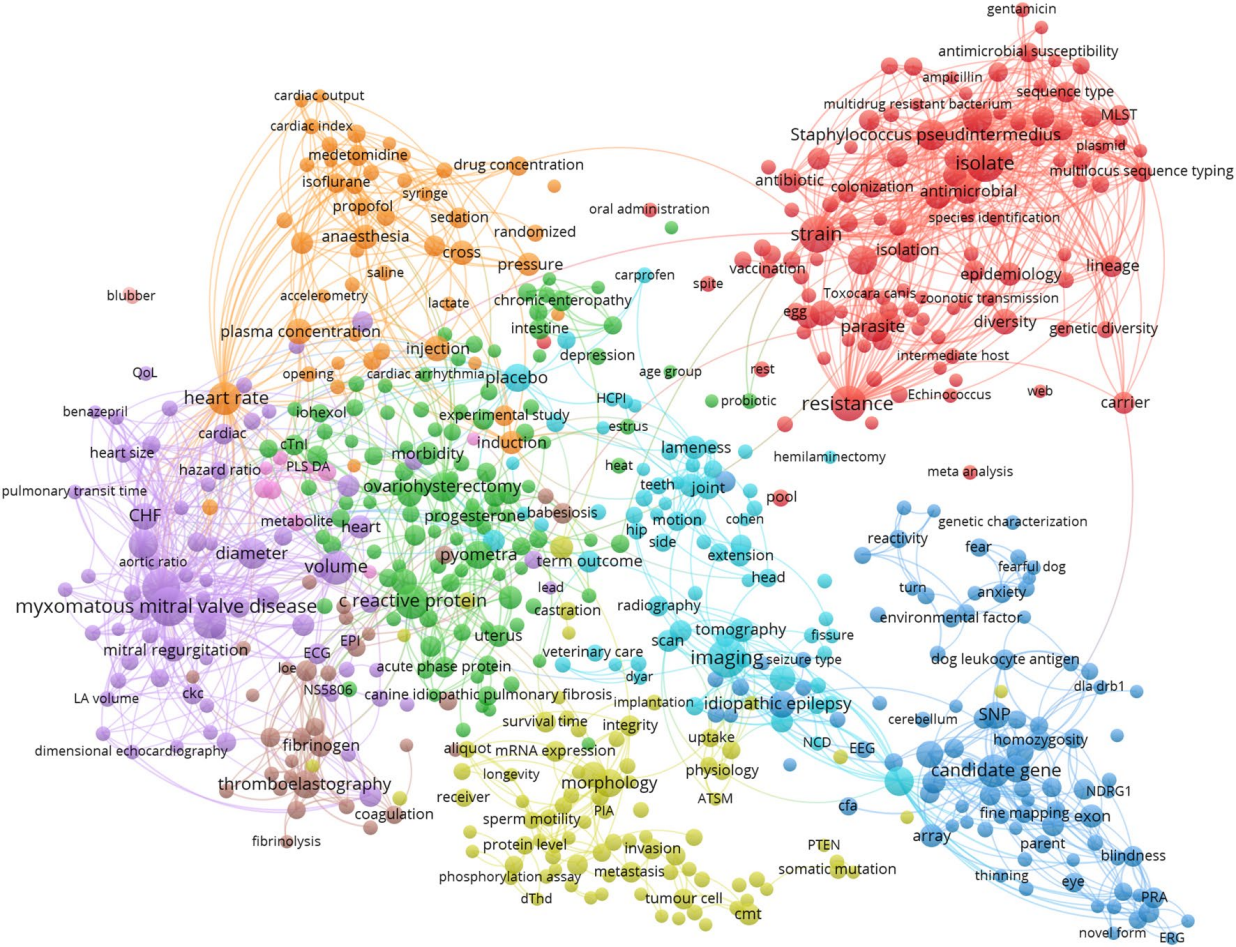
Term maps of the fields of clinical research on dogs in the Nordic countries. The networks are based on term co-occurrences in the titles and abstracts of published clinical articles. The distance between two terms in a map reflects the relatedness of the terms, and colours represent clusters of related terms. The networks were created from terms occurring in a minimum of four articles. The 60% most relevant terms, based on their VOSviewer relevance score, were included in the maps (n = 635). Links are shown for pairs of terms which co-occur in at least three articles

**Fig. 4:**
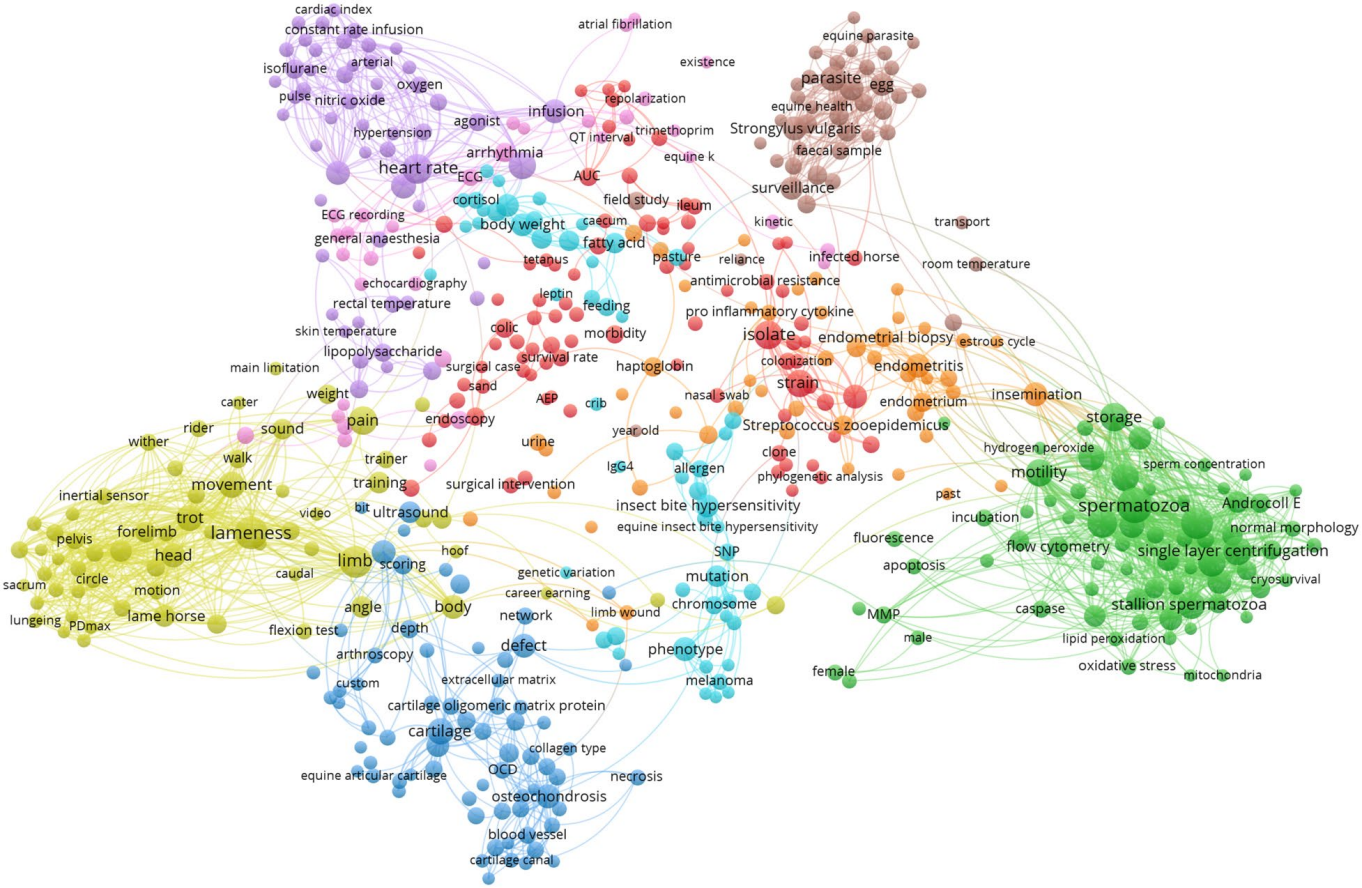
Term maps of the fields of clinical research on horses in the Nordic countries. The networks are based on term co-occurrences in the titles and abstracts of published clinical articles. The distance between two terms in a map reflects the relatedness of the terms, and colours represent clusters of related terms. The networks were created from terms occurring in a minimum of four articles. The 60% most relevant terms, based on their VOSviewer relevance score, were included in the maps (n = 520). Links are shown for pairs of terms which co-occur in at least three articles

Canine visualized research areas included anaesthesiology, cardiology, clinical pathology, genetics, diagnostic imaging, infectious diseases, neurology, oncology, orthopaedics, reproduction and surgery (Fig. 2). Feline research areas included cardiology, clinical pathology, diagnostic imaging, infectious diseases, metabolic diseases, reproduction, urinary tract diseases and surgery (Fig. 3). Visualized research areas for horses were anaesthesiology, cardiology, genetics, infectious diseases, metabolic diseases, orthopaedics, pathology, reproduction and surgery (Fig. 4).

### Preferred scientific journals

Studies in cats and horses were most commonly published in a journal focusing on the actual species, whereas it was a broader species scope in the top journal for canine studies (Table 3). The second most popular journal for each of the species was Acta Veterinaria Scandinavica. Several publications were in journals with a wide scope, but there were also discipline-oriented journals among the ten journals that featured the most articles. For studies in dogs, these were journals focusing on internal medicine, clinical pathology, oncology and reproduction; for cats, medicine and surgery, clinical pathology, reproduction, cardiology and emergency and critical care; and for horses, they covered veterinary education, reproduction, parasitology and internal medicine (Table 3). Among the ten journals with the highest number of published articles on horses, three were exclusively dedicated to equine studies.

**Table 3:** Journals with most publications on clinical veterinary research concerning dogs, cats and horses 2010–2019

Publications on cats		Publications on dogs		Publications on horses	
Name	n	Name	n	Name	n
Journal of Feline Medicine and Surgery	42	Journal of Veterinary Internal Medicine	101	Equine Veterinary Journal	79
Acta Veterinaria Scandinavica	18	Acta Veterinaria Scandinavica	94	Acta Veterinaria Scandinavica	55
Journal of Veterinary Internal Medicine	17	Veterinary Journal	70	Veterinary Journal	42
Veterinary Clinical Pathology	12	PLOS One	61	Journal of Equine Veterinary Science	36
Theriogenology	8	BMC Veterinary Research	43	Equine Veterinary Education	36
Veterinary Journal	8	Veterinary Clinical Pathology	31	Reproduction In Domestic Animals	24
Journal of Veterinary Cardiology	7	Veterinary Record	26	Theriogenology	22
Reproduction in Domestic Animals	7	Journal of Small Animal Practice	22	Veterinary Parasitology	21
Journal of Veterinary Emergency and Critical Care	7	Veterinary and Comparative Oncology	21	American Journal of Veterinary Research	20
BMC Veterinary Research	6	Theriogenology	21	Journal of Veterinary Internal Medicine	20

### Doctoral theses on cats, dogs and horses 2010–2019

In total, 970 PhD theses were published by Nordic veterinary faculties from 2010 to 2019. Of these, 178 (18%) concerned cats, dogs, horses or a combination of these species (Table 4). The highest proportion of doctoral theses on cats, dogs and horses in relation to other species was produced by SLU, followed by UH and UCPH. At NMBU, only 10% of theses from the veterinary faculty focused on cats, dogs and/or horses. For UCPH and UH, most of these theses concerned dogs (46% and 64%, respectively), followed by horses (26% and 22%) and cats (11% and 6%). For SLU, most theses (49%) concerned horses, followed by dogs (38%) and cats (11%). For NMBU, the number of theses was very similar for dogs and horses (46% and 43%, respectively) whereas only one thesis (3.5%) concerned cats only.

**Table 4:** Number of PhD theses concerning the species cats, dogs and horses, all scientific areas

University	Total number	Cats/dogs/ horses (% of total^a^ %)	Cats only	Dogs only	Horses only	Combination of species^b^
NMBU	268	28 (10)	1	13	12	2
SLU	258	65 (25)	7	25	32	1
UCPH	192	35 (18)	4	16	9	6
UH	255	50 (20)	3	32	11	4
Total	973	178 (18)	15	86	64	13

Number of PhD theses from *NMBU* (Faculty of Veterinary Medicine), *SLU* (Faculty of Veterinary Medicine and Animal Science), *UCPH* (Departments of Veterinary and Animal Sciences and of Veterinary Clinical Sciences), and *UH* (Faculty of Veterinary Medicine)

^a^PhD theses on cats, dogs and horses related to the total number of PhD theses

^b^PhD theses including more than one of the species cats, dogs, horses

### Human resources

**Fig. 5:**
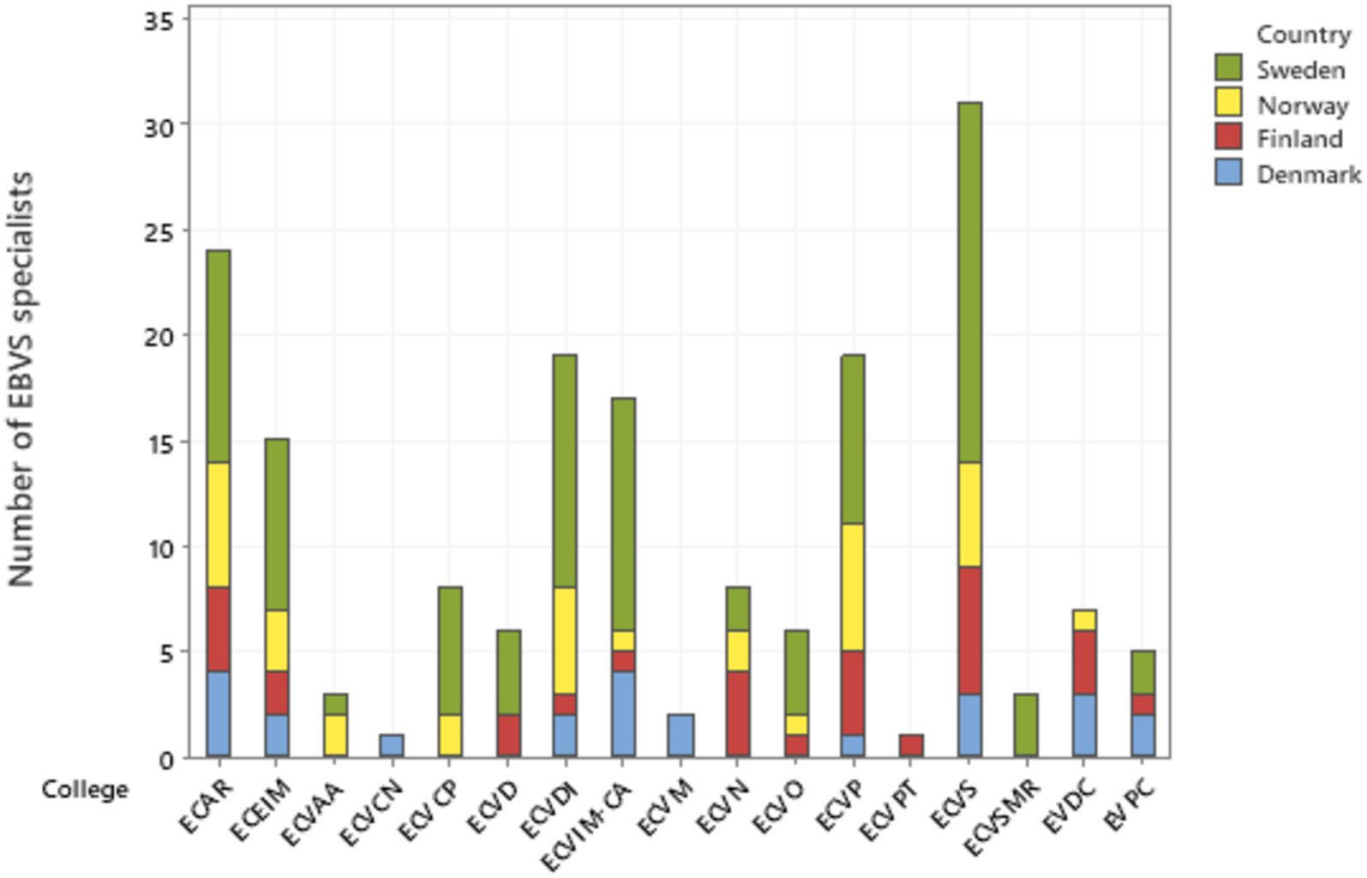
Number of EBVS^™^ specialists from the different countries within European colleges. The number of European Veterinary Specialists^™^ in colleges that were most closely linked to clinical research in cats, dogs and horses is shown. Defined as most closely linked to clinical research in cats, dogs and horses were European Colleges of: *ECAR* Animal Reproduction, *ECEIM* Equine Internal Medicine, *ECVAA* Veterinary Anaesthesia and Analgesia, *ECVCN* Veterinary and Comparative Nutrition, *ECVCP* Veterinary Clinical Pathology, *ECVD* Veterinary Dermatology, *ECVDI* Veterinary Diagnostic Imaging, *ECVIM-CA* Veterinary Internal Medicine-Companion Animals, *ECVN* Veterinary Neurology, *ECVO* Veterinary Ophthalmologists, *ECVP* Veterinary Pathologists, *ECVCP* Veterinary Pharmacology and Toxicology, *ECVS* Veterinary Surgeons, *ECVSMR* Veterinary Sports Medicine and Rehabilitation, and *EVDC* Veterinary Dental College, and EVPC: European Veterinary Parasitology College

There were 252 Danish, Finnish, Norwegian and Swedish European EBVS^®^ Veterinary Specialists in 2021. When compared to the total number of European Veterinary Specialists^™^ in the country, the percentage of European Veterinary Specialists^™^ of colleges most closely linked to clinical research in cats, dogs and horses was 42% (20/48) for Denmark, 74% (29/39) for Finland, 76% (34/55) for Norway and 74% (81/110) for Sweden. For a distribution between the different colleges, see Fig. 5.

In total, there were 27 professors and 55 associate professors (docents) working with companion animals and equine research in January 2021 in the veterinary clinical departments in Finland, Denmark, Sweden and Norway. They were distributed as follows: NMBU: 2 professors and 15 associate professors; SLU: 9 professors and 14 associate professors; UCPH: 8 professors and 14 associate professors and in addition 4 professors and 5 associate professors working cross species; and UH, 4 professors and 7 associate professors.

### Funding of veterinary clinical research in cats, dogs and horses

The top funding bodies for clinical research on cats and dogs in Denmark were Independent Research Fund Denmark, Agria and SKK Research Fund, Royal Canin, and Antech Imaging Systems, and for horses: Independent Research Fund Denmark, Horse Levy Foundation, Kustos, Xintela and Antech Imaging Systems.

In Finland, the most important funding bodies for dogs and cats were Academy of Finland, Agria and the Finnish Kennel Club Research Fund, The American Kennel Club Canine Health Foundation (USA), Nylands nation, Svenska Kulturfonden, Finnish Veterinary foundations and pharmaceutical companies, and for horses: the Academy of Finland, Finnish Veterinary Foundations and pharmaceutical companies.

In Sweden, the most important funding bodies for dogs and cats were The Greater Stockholm Veterinary Care Foundation (Stiftelsen Stor-Stockholms djursjukhus), Agria and The Swedish Kennel Club Research foundation, research foundations administered by SLU, Thure F and Karin Forsberg’s foundation, Jan Skogsborg’s foundation, and pharmaceutical companies, and for horses: The Swedish-Norwegian Foundation for Equine Research (Stiftelsen Hästforskning) and Formas (a Swedish government research council for sustainable development).

In Norway, the Ministry of Education and Research and the Swedish-Norwegian Foundation for Equine Research (Stiftelsen Hästforskning) were the most important funding bodies. In addition, there were nongovernmental bodies such as Agria, Norwegian Veterinary foundations, Stiftelsen forskningsfondet kreft hos hund and private memorial funds.

During 2010–2019, Formas funded 30 clinical veterinary research projects on sports and companion animals in the annual open call. The success rate for applications for the different species varied between 5% (cats) and 14% (a combination of more than one of the species) (Table 5). The overall success rate from Formas during the period was 14%.

**Table 5:** Outcome for applications to Formas annual open call (Sweden) 2010–2019

**Cat**	**19**
Rejected	18
Granted	1 (5%)
**Dog**	**97**
Rejected	84
Refused by Formas	1
Granted	12 (12%)
**Horse**	**135**
Rejected	118
Refused by Formas	3
Granted	14 (10%)
**Multiple species**	**22**
Rejected	18
Withdrawn by author	1
Granted	3 (14%)
**Total granted concerning dogs,** **cats or horses**	**30/273 (11%)**

Numbers in bold describe the total number of applications for the respective species. The percentage granted applications out of the total number of grant applications for that specific species is given within parentheses. The overall percentage of granted applications from Formas during the period was 14%

**Fig. 6:**
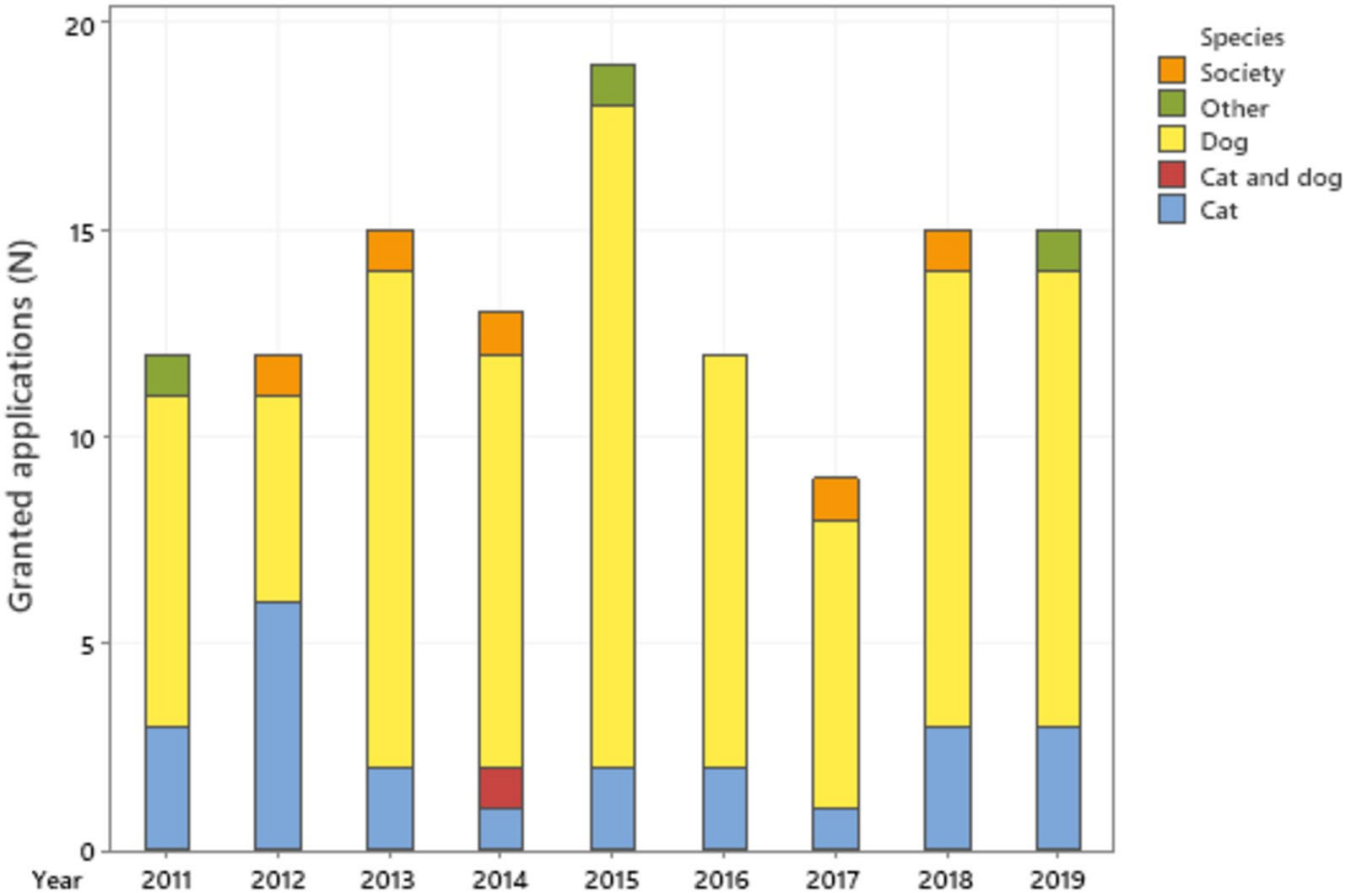
Number of granted applications from the SKK and Agria Research Fund. Number of granted applications years 2011–2019 for projects concerning cats, dogs, both species, “other”: (rabbits or guinea pigs) or “society”: projects on different benefits of cats or dogs for society

The SKK and Agria research fund granted 122 projects in total during 2011–2019, with 9–19 projects per year. Most projects (74%, n = 90) concerned dogs, 19% (n = 23) concerned cats and 0.8% (n = 1) both cats and dogs (Fig. 6). Four percent of the projects (n = 5) concerned the value of companion animals for humans or for society, and 2% (n = 3) concerned rabbits or guinea pigs. The grants were distributed to the main applicants affiliated with 18 different organizations, most to SLU (n = 45), UCPH (n = 33) and NMBU (n = 17). Most granted projects had applicants from Sweden (n = 65), followed by Denmark (n = 34), Norway (n = 18) and Finland (n = 3). Two projects were collaborations between Sweden and Denmark and between Sweden and Norway. Most grants were for 1-year projects (n = 43), followed by 2- or 3-year projects (n = 39 for both). Information on duration was missing for one project.

The Agria and SKK Research Fund has a two-step process for research applications. According to the research fund, the fund receives 50–75 applications in the first step, and approximately 2/3 of these are rejected. Approximately 80% of the 20–25 applications in the second step were granted, leading to an approximate overall acceptance rate of 25%.

### Household costs

**Fig. 7:**
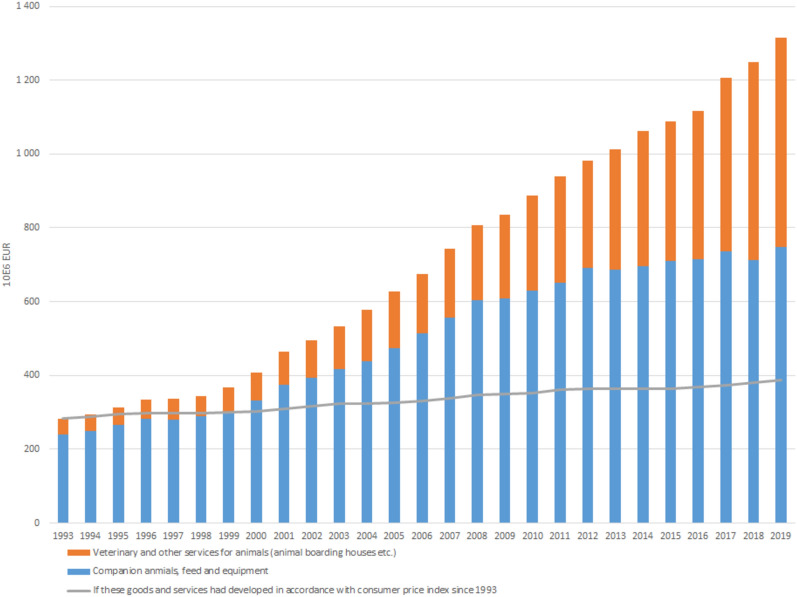
Costs for veterinary and other services for animals and for companion animals, their feed and equipment in Sweden 1993–2019. The line shows what the costs would have been if they had followed the consumer price index since 1993. Data from Statistics Sweden (SCB)

Household costs for veterinary and other services for dogs and cats in Sweden increased 121%, from 2 905 000 000 SEK in 2010 to 6 407 000 000 in 2019. The costs for the animals, their feed and equipment increased 19%, from 7 118 000 000 SEK in 2010 to 8 449 000 000 SEK in 2019 (Fig. 7). As can be seen in Fig. 7, from 2000 onward these costs increased far beyond the consumer price increase.

## Discussion

From 2010 to 2019, the output of canine, feline and equine clinical research across the Nordic countries amounted to more than 150 publications per year, the majority from the four universities with veterinary education. During the same period, costs for veterinary and other services steadily increased. The growth of the companion animal sector can be expected to drive an increased demand for veterinary services, necessitating clinical research to develop new treatments, products, and technologies. However, the expansion of the veterinary healthcare sector, as shown in Fig. 7, did not correspond to a rise in the number of clinical research publications, which remained stagnant after 2014 (Fig. 1). While there typically exists a delay of 2 years or more between funding and publication, this stagnation suggests that research funding has not kept pace. Given the significant societal and economic importance in the field, there is a compelling case for revising the approach to funding to stimulate research within the area.

Because there was only one university in each Nordic country educating veterinarians during the study period, except for Iceland, which does not have a veterinary medicine programme, promoting collaboration between them may be an efficient way to increase the critical mass within the different clinical research areas. Seven percent of the publications had authors from more than one Nordic university, with the most productive collaborations being between UCPH and SLU and between NMBU and SLU. The universities are also important research partners for private organizations; 68% of the publications on dogs, cats and horses that had authors from the two largest private organizations were authored in collaboration with at least one of the universities. The different Nordic countries have a similar view of animals, research culture, a tradition for evidence based veterinary care, and a similar tradition of restrictive use of antibiotics, which is beneficial for clinical collaborations. The universities contributing with the largest number of publications within the area, SLU and UCPH, produced 14–15% of their publications in collaboration with one of the other universities, and for NMBU, the corresponding figure was 28%. The Nordic universities that educate veterinarians were important collaborators for veterinary clinical research, but SLU, UCPH and NMBU all had additional collaborations with another national university among their top three collaborators. All Nordic universities had at least one international collaborator among their top three except for UH, whose top three collaborators were national. In a relatively small field, it should be kept in mind that individuals or groups with unusually high output may largely influence the results.

The number of articles and doctoral theses was highest for dogs and lowest for cats. The number of dogs is higher than the number of horses, but there are more cats than both dogs and horses, and this is not reflected in the number of published papers. There are several possible explanations for this. Traditionally, the perceived value of a cat has been lower, as shown by a considerably lower proportion of insured cats compared to dogs and horses [34, 35]. Additionally, the demand for advanced veterinary care for cats has historically been lower than for dogs [32]. This perception may also be mirrored in the underdeveloped field of feline research and the very low proportion of successful funding applications for cat-related studies submitted to the Swedish Research Council Formas. However, other factors could also be contributing to this trend. The economic values of individual horses and the horse industry are larger than the respective figures for cats or dogs [40]. The equine research funding collaboration between Norway and Sweden, with both private and governmental contributions, possibly enables the granting of larger projects. No equivalent to this research funding body has been made available for canine and feline research. However, as many veterinary visits concern cats, clinical research for better veterinary care of cats is an area with potential for development.

Cats, dogs and horses are highly treasured, and their economic value includes both use and non-use values, i.e. values for people that come in direct contact with the animals and people that do not but still consider their value [1, 2, 40]. Despite their value and the large-sized animal healthcare sector, the mean proportion of PhD theses concerning these animals was only 18% of all PhD theses at the veterinary faculties or corresponding departments at the Nordic universities. The proportion varied between the universities, from 10% at NMBU to 25% at SLU. Several factors affect research topics for PhD theses, including the teaching and clinical service workload for faculty that work clinically compared to those that do not. In addition, the research interest of the academic staff, tradition, and especially the limited possibility to achieve full external funding for salaries of PhD students are at play. At NMBU, the percentage of time delegated to research for the staff has been described as low compared to other veterinary universities [48], which may contribute to the low number of theses concerning cats, dogs and horses. A survey at UCPH describes a similar situation. Finding funding for PhD students is a challenge. For Denmark and Finland, the proportion of doctoral theses for the different species mirrors the proportion of publications within these countries. In Denmark the number of scholarships funded by the university has decreased. Interestingly, in Norway and Sweden, the proportion of doctoral theses concerning horses was comparatively higher. At SLU, there were more theses concerning horses than dogs, and at NMBU, the numbers for the species were similar. This is crucial to consider because a doctoral thesis, comprising a series of studies focusing on a specific area, both serves as the cornerstone of a developing researcher's scientific education and enables a more comprehensive exploration and generation of research outcomes within a scientific domain. However, it is important to note that the translation of these findings into clinical implications may have a considerable turnaround time. Although there may be other reasons for the relatively high number of PhD theses on horses at NMBU and SLU, the Swedish-Norwegian Foundation for Equine Research most likely has had a strong positive impact. Larger governmental funding bodies, such as Formas in Sweden, are important sources for funding PhD students and postdocs within several scientific areas and for all domesticated species. For applications concerning cats, dogs, and horses as single species, the success rate was lower than average for Formas open calls. Several criteria are assessed by Formas: novelty and originality, scientific approach, societal value, work plan and competence. It was not within the scope of the present study to evaluate the assessments of these individual criteria, but such an evaluation would be valuable to enable directed measures for increased success rate, at least from this specific fund.

To develop all aspects of companion animal and equine veterinary care, research is key. The sector considers a lack of veterinarians and veterinary nurses as a major obstacle for continuing development [33]. Teaching, clinical and administrative work makes up a large proportion of the workload within academia, and in Norway, the limited time for research for personnel working at NMBU has been highlighted [48]. Funding for salaries is thus needed not only for PhD students but also after the PhD defence. For postdoctoral research activities, research tasks can be a limited part of the workload, together with clinical work, teaching and administrative duties. Unfortunately, many of the most important funding bodies for clinical research, especially for dogs and cats, generally do not cover costs for salaries but consider funding of running costs as a better value-for money. Consequently, salary for the researcher must be sought from other sources. The situation is similar in the UK, where research centring canine health and welfare largely rely on canine specific funding, stressing the importance of strategically directing such funding as effectively as possible [49]. The overhead costs of the universities are considered high by some funding bodies that as a consequence fund overhead costs to a lower degree, making it difficult to fully finance the studies. Within academia, there is a high demand and competition for external funding, and the situation is similar for veterinarians interested in performing research while in clinical practice. Except for those within a residency programme, who generally will have time allotted for education, including their research projects, veterinary practitioners will generally not have time dedicated for research. Several residency programmes are performed at university clinics. However, the number of European Veterinary Specialists^™^ is not related to the research output of a country; for example, Denmark, with the second highest publication output, has the lowest number of European Veterinary Specialists^™^. European Veterinary Specialists^™^ are a prerequisite for residency training, and residents perform research studies within their education, but their relative contribution to the research output within the Nordic countries thus seems limited.

Academic staff is a prerequisite for PhD education. There was a similar number of professors and associate professors focused on dogs, cats and horses in UCPH and SLU and approximately half as many in UH, but the number of PhD theses within these species was much higher at UH than at UCPH. Simple counting of professors and associate professors is a very blunt tool for comparing human resources because the time allocated for research may vary heavily between persons. The results indicate that the possibility for professors to obtain internal or external financing for PhD students and other research projects, and the amount of time for research within their positions, are better associated with research output than their sole number.

For all three animal species, research was published in both topic-oriented and general scientific journals. The value of a Nordic scientific journal is reflected by the fact that Acta Veterinaria Scandinavica is the second most used journal for all three species. Some scientific areas, such as cardiology, infectious diseases, reproduction and surgery, were prominent for all three species, whereas pathology was only prominent for horses, neurology and oncology were only prominent for dogs, and urinary tract diseases were only prominent for cats. For certain areas, interspecies collaborations may thus be more fruitful, whereas for others, collaborations between disciplines may have a greater value. Veterinary clinical research in Nordic countries is broad, with several prominent scientific areas, constituting a possible basis for collaboration. The fact that several areas are internationally prominent and competitive for more than one species also emphasizes the possibility for comparative studies.

The majority of veterinarians in the Nordic countries work in clinical practice, predominantly companion animal clinical practice [50]. Research funding is a prerequisite not only for the development and advancement of the field, but also for increasing the competence of the faculty and staff, ensuring an increase in the evidence-based provision of healthcare to the animals and thus increasing animal welfare and quality of life. Research funding is also a driver for veterinarians to stay within the sector—a sector desperately needing more animal healthcare personnel.

## Conclusion

Despite a high societal value of the species, veterinary clinical research on sports and companion animals has not increased, in contrast to the veterinary healthcare sector. Activities stimulating the research area, e.g. funding bodies enabling coverage of salaries, are needed. The development of Nordic veterinary clinical care may benefit from strengthened cooperation between countries.

## Data Availability

The datasets supporting the conclusions of this article are available in the Swedish National Data Service at: https://doi.org/10.5878/8cng-q629.

## References

[1] Hoffmann R, Lagerkvist CJ, Hagberg Gustavsson M, Holst BS. Economic perspective on the value of cats and dogs. Soc Anim. 2018;27:595–613. 10.1163/15685306-12341494.

[2] Hoffmann R, Lagerkvist CJ, Hagberg Gustavsson M, Holst BS. An empirical examination of the conceptualization of companion animals. BMC Psychol. 2018;6:15. 10.1186/s40359-018-0228-1. 29724234 10.1186/s40359-018-0228-1 PMC5934865

[3] Hoffmann R, Lokrantz M, Lagerkvist CJ. Hagberg Gustavsson M, Holst BS. Värdet av hundar och katter i Sverige. SLU Framtidens djur, natur och hälsas rapportserie. Uppsala 2017. p. 62.

[4] Bunketorp-Käll L, Lundgren-Nilsson Å, Samuelsson H, Pekny T, Blomvé K, Pekna M, et al. Long-term improvements after multimodal rehabilitation in late phase after stroke: a randomized controlled trial. Stroke. 2017;48:1916–24. 10.1161/strokeaha.116.016433. 28619985 10.1161/STROKEAHA.116.016433

[5] Coffman AN, Bernstein ER, Davies SC, Justice AF. The impact of a canine-assisted reading program on readers needing extra practice. Read Teach. 2023;76:724–34. 10.1002/trtr.2192.

[6] Grajfoner D, Ke GN, Wong RMM. The effect of pets on human mental health and wellbeing during COVID-19 lockdown in Malaysia. Animals. 2021;11(9):2689. 34573655 10.3390/ani11092689 PMC8470955

[7] Harris A, Williams JM. The impact of a horse riding intervention on the social functioning of children with autism spectrum disorder. Int J Environ Res Public Health. 2017;14:776. 10.3390/ijerph14070776. 28708075 10.3390/ijerph14070776 PMC5551214

[8] Lindström Nilsson M, Funkquist EL, Edner A, Engvall G. Children report positive experiences of animal-assisted therapy in paediatric hospital care. Acta Paediatr. 2020;109:1049–56. 10.1111/apa.15047. 31597211 10.1111/apa.15047

[9] Lönker NS, Fechner K, Wahed AAE. Horses as a crucial part of One Health. Vet Sci. 2020;7:28. 10.3390/vetsci7010028. 32121327 10.3390/vetsci7010028 PMC7157506

[10] Lundquist WP. Disability, riding, and identity: a qualitative study on the influence of riding on the identity construction of people with disabilities. Intl J Disabil Dev Educ. 2014;61:67–79. 10.1080/1034912X.2014.878543.

[11] Martin F, Bachert KE, Snow L, Tu H-W, Belahbib J, Lyn SA. Depression, anxiety, and happiness in dog owners and potential dog owners during the COVID-19 pandemic in the United States. PLoS ONE. 2021;16: e0260676. 10.1371/journal.pone.0260676. 34910761 10.1371/journal.pone.0260676 PMC8673598

[12] Mičková E, Machová K, Daďová K, Svobodová I. Does dog ownership affect physical activity, sleep, and self-reported health in older adults? Int J Environ Res Public Health. 2019;16:3355. 10.3390/ijerph16183355. 31514379 10.3390/ijerph16183355 PMC6765935

[13] Mubanga M, Byberg L, Nowak C, Egenvall A, Magnusson PK, Ingelsson E, et al. Dog ownership and the risk of cardiovascular disease and death—a nationwide cohort study. Sci Rep. 2017;7:15821. 10.1038/s41598-017-16118-6. 29150678 10.1038/s41598-017-16118-6 PMC5693989

[14] Pálsdóttir AM, Gudmundsson M, Grahn P. Equine-assisted intervention to improve perceived value of everyday occupations and quality of life in people with lifelong neurological disorders: a prospective controlled study. Int J Environ Res Public Health. 2020;17:2431. 10.3390/ijerph17072431. 32260047 10.3390/ijerph17072431 PMC7177295

[15] Jia L, Yang X, Jiang Y. The pet exposure effect: exploring the differential impact of dogs versus cats on consumer mindsets. J Mark. 2022;86:42–57. 10.1177/00222429221078036.

[16] Kristiansen JE. Kjaeledyr i norske husholdninger. Samfunnsspeilet 1994. p. 18–21.

[17] Lounasmeri L. Eläinruoka on rakkaudella ostettu Sparraaja. 2002;4:34–8.

[18] Nielsen B. Familiernes kæledyr. 2000. Nyt fra Danmarks statistik Nr. 499, https://www.dst.dk/pukora/epub/nyt/2000/nr499.pdf

[19] Hundar och katter allt vanligare i svenska hem. 2017. https://novus.se/nyheter/2017/11/hundar-och-katter-allt-vanligare-svenska-hem/. Accessed 09 May 2022.

[20] Amiot CE, Gagné C, Bastian B. Pet ownership and psychological well-being during the COVID-19 pandemic. Sci Rep. 2022;12:6091. 10.1038/s41598-022-10019-z. 35413973 10.1038/s41598-022-10019-z PMC9002031

[21] Hytönen MK, Lohi H. Canine models of human rare disorders. Rare Dis. 2016. 10.1080/21675511.2016.1241362. 27803843 10.1080/21675511.2016.1241362 PMC5070630

[22] Shearin AL, Ostrander EA. Leading the way: canine models of genomics and disease. Dis Model Mech. 2010;3:27–34. 10.1242/dmm.004358. 20075379 10.1242/dmm.004358 PMC4068608

[23] Kol A, Arzi B, Athanasiou KA, Farmer DL, Nolta JA, Rebhun RB, et al. Companion animals: translational scientist’s new best friends. Sci Transl Med. 2015. 10.1126/scitranslmed.aaa9116. 26446953 10.1126/scitranslmed.aaa9116 PMC4806851

[24] Fall T, Lundholm C, Örtqvist AK, Fall K, Fang F, Hedhammar Å, et al. Early exposure to dogs and farm animals and the risk of childhood asthma. JAMA Pediatr. 2015;169: e153219. 10.1001/jamapediatrics.2015.3219. 26523822 10.1001/jamapediatrics.2015.3219

[25] Chandler M, Cunningham S, Lund EM, Khanna C, Naramore R, Patel A, et al. Obesity and associated comorbidities in people and companion animals: a one health perspective. J Comp Pathol. 2017;156:296–309. 10.1016/j.jcpa.2017.03.006. 28460795 10.1016/j.jcpa.2017.03.006

[26] Ojwang V, Nwaru BI, Takkinen HM, Kaila M, Niemelä O, Haapala AM, et al. Early exposure to cats, dogs and farm animals and the risk of childhood asthma and allergy. Pediatr Allergy Immunol. 2020;31:265–72. 10.1111/pai.13186. 31829464 10.1111/pai.13186

[27] Lappalainen MH, Huttunen K, Roponen M, Remes S, Hirvonen MR, Pekkanen J. Exposure to dogs is associated with a decreased tumour necrosis factor-α-producing capacity in early life. Clin Exp Allergy. 2010;40:1498–506. 10.1111/j.1365-2222.2010.03566.x. 20633030 10.1111/j.1365-2222.2010.03566.x

[28] Kantele A, Paajanen J, Turunen S, Pakkanen SH, Patjas A, Itkonen L, et al. Scent dogs in detection of COVID-19: triple-blinded randomised trial and operational real-life screening in airport setting. BMJ Glob Health. 2022;7: e008024. 10.1136/bmjgh-2021-008024. 35577391 10.1136/bmjgh-2021-008024 PMC9108438

[29] St.meld. nr. 12. (2002–2003) Om dyrehold og dyrevelferd. 2003. p. 185.

[30] Wallis LJ, Szabó D, Erdélyi-Belle B, Kubinyi E. Demographic change across the lifespan of pet dogs and their impact on health status. Front Vet Sci. 2018;5:200. 10.3389/fvets.2018.00200. 30191153 10.3389/fvets.2018.00200 PMC6115627

[31] Cozzi B, Ballarin C, Mantovani R, Rota A. Aging and veterinary care of cats, dogs, and horses through the records of three university veterinary hospitals. Front Vet Sci. 2017;4:14. 10.3389/fvets.2017.00014. 28261586 10.3389/fvets.2017.00014 PMC5306394

[32] Springer S, Sandøe P, Grimm H, Corr SA, Kristensen AT, Lund TB. Managing conflicting ethical concerns in modern small animal practice-a comparative study of veterinarian’s decision ethics in Austria. Denmark and the UK PLoS One. 2021;16: e0253420. 10.1371/journal.pone.0253420. 34143850 10.1371/journal.pone.0253420 PMC8213188

[33] Svensk Djursjukvård branschrapport 2019. p. 15. https://www.grona.org/svensk-djursjukvard/svensk-djursjukvards-branschrapport/

[34] Nordqvist L, Adamsson J. 2018. Bättre konkurrens om fler byter djurförsäkring. Konkurrensverkets rapportserie p. 100. https://www.konkurrensverket.se/globalassets/dokument/informationsmaterial/rapporter-och-broschyrer/rapportserie/rapport_2018-6.pdf

[35] Richvoldsen G. 2021. Mange dropper hundeforsikring. https://kommunikasjon.ntb.no/pressemelding/mange-dropper-hundeforsikring?publisherId=14507710&releaseId=17899390. Accessed 9 May 2022.

[36] Bederoff J: Fyrfota kunder allt fler i coronatider. 2020. https://www.di.se/nyheter/fyrfota-kunder-allt-fler-i-coronatider/. Accessed 13 July 2023.

[37] Veterinæren : yrke - organisasjon - samfunn : Den norske veterinærforening 125 år. Den norske veterinærforening. 2013.

[38] Dyreklinikk.no Landets dyreklinikker på nett. https://www.dyreklinikk.no/ Accessed 10 May 2022.

[39] Hästar och anläggningar med häst. Resultat från en intermittent undersökning. Sveriges officiella statistik Statistiska meddelanden. 2016;2017:23.

[40] Hästnäringen i siffror. 2019. https://hastnaringen-i-siffror.se/. Accessed 10 May 2022.

[41] Enhäll J, Nordgren M, Kättström H. Hästhållning i Sverige 2010. Swedish Board of Agriculture; 2012. p. 72.

[42] Association ST. 2022. Swedish Trotting Association. Yearly report on racing. https://www.travsport.se/siteassets/relaterade-dokument/nyheter/2023/svensk-travsport-arsrapport_tavling_2022-230213.pdf?269. Accessed 29 October 2023.

[43] Swedish Equestrian Federation. https://www.ridsport.se/Omoss/Kontaktaoss/SwedishEquestrianFederation Accessed 10 May 2022.

[44] Riksidrottsförbundet: Idrottsrörelsen i siffror. 2023. https://www.rf.se/forskning-och-statistik/statistik/idrottsrorelsen-i-siffror. Accessed 29 October 2023.

[45] van Eck NJ, Waltman L. Software survey: VOSviewer, a computer program for bibliometric mapping. Scientometr. 2010;84:523–38. 10.1007/s11192-009-0146-3. 10.1007/s11192-009-0146-3 PMC2883932 20585380

[46] van Eck NJ, Waltman L. Text mining and visualization using VOSviewer. ISSI Newsletter. 2011;7(3):50–4.

[47] European Board of Veterinary Specialisation. https://ebvs.eu Accessed 1 Feb 2021.

[48] Akselsen B. Fra klinikkene på Adamstua til Universitetsdyrehospital på Ås - A til Å rapporten. 2010. p. 103.

[49] Skipper AM, Packer RMA, O’Neill DG. Researcher, research thyself? Mapping the landscape of canine health and welfare research funding provided by UK not-for-profit organisations from 2012–2022. PLoS ONE. 2024;19: e0303498. 10.1371/journal.pone.0303498. 38781269 10.1371/journal.pone.0303498 PMC11115267

[50] FVE. Vet Survey. Survey of the veterinary profession in Europe: Federation of veterinarians of Europe; 2019. https://fve.org/cms/wp-content/uploads/FVE-Survey-2023_updated-v3.pdf

